# The Brain and Obesity Lectures Series – the beginning of a new field?

**DOI:** 10.1111/j.1749-6632.2012.06721.x

**Published:** 2012-08-08

**Authors:** Giovanni Cizza, Kristina I Rother

**Affiliations:** 1 Section on Neuroendocrinology of Obesity, NIDDK, National Institutes of Health BethesdaMaryland; 2 Section on Pediatric Diabetes and Metabolism, NIDDK, National Institutes of Health BethesdaMaryland

It is a great pleasure to introduce the Brain and Obesity Lecture Series in this issue of *Annals of the New York Academy of Sciences*. These lectures were initiated in 2006 when the Intramural Center for Obesity was about to open at the National Institutes of Health (NIH) Clinical Center, a state-of-the art facility that includes a 21-bed inpatient facility and three metabolic chambers and other equipment for studies on phenotyping subjects with obesity. An interdisciplinary group of clinical investigators was recruited that included endocrinologists, nutritionists, geneticists, exercise physiologists, and neuropsychologists. The fact that the Intramural Center for Obesity was part of the NIH Clinical Center, a 240-bed facility completely devoted to clinical research, offered a valuable opportunity for collaboration and cross-fertilization within clinical research and with translational and basic research.

We shared with other colleagues, mostly in the field of neurosciences, the conviction that the brain plays a pivotal role in obesity. This notion, largely accepted today, was quite controversial at the time. Rather, it was thought that obesity was a problem of eating too much and exercising too little. Without challenging this obvious tenet—derived from the law of thermodynamics—we thought that there was much more to it. Another commonly held notion was that “a calorie, is a calorie, is a calorie,” a notion that has now been revisited. The Brain and Obesity Lecture Series started us on a journey of exploration, a search for ongoing work based on the idea that the brain had something to do with the development of obesity. In the following five years, there were a total of 16 lectures (Table 1). On March 22, 2012, ten of the original speakers, or members of their respective teams, returned to the NIH Clinical Center for a final round of talks and discussions. What is summarized below is the distillate of those lectures.

**Table 1:** Brain and Obesity Lecture series hosted by the NIH Clinical Center between 2007 and 2012

Speaker	Date	Affiliation	Title
Saverio Cinti	October 11, 2007	University of Ancona, Italy	Transdifferentiation properties of the adipose organ
Sabrina Diano	January 30, 2008	Yale School of Medicine, New Haven, Connecticut	Ghrelin's role in appetite and memory
Mark Mattson	February 20, 2008	National Institute on Aging, Baltimore, Maryland	BDNF as a regulator of systemic and brain energy metabolism
Marco Boscaro	April 2, 2008	University of Ancona, Italy	Visceral adipose tissue: emerging role of gluco- and mineralocorticoid hormones
Catherine M. Kotz	December 3, 2008	University of Minnesota, Minneapolis, Minnesota	Brain mechanisms underlying nonexercise activity thermogenesis and obesity
Luigi Fontana	December 10, 2008	Washington University School of Medicine, St Louis, Missouri	Adiposity, calorie restriction, and aging
David Gozal	February 25, 2009	University of Chicago, Illinois	Sleep deprivation and the obesity epidemic in children
Silvana Obici	March 25, 2009	University of Cincinnati, Ohio	Hypothalamic control of glucose secretion
Eve Van Cauter	April 9, 2009	University of Chicago, Illinois	Metabolic and endocrine consequences of sleep deprivation
William A. Banks	May 20, 2009	University of Washington, Seattle, Washington	Role of the blood–brain barrier in the control of food intake and body weight regulation
Stephen C. Benoit	November 4, 2009	University of Cincinnati, Ohio	Learned and cognitive controls of ingestive behavior
Renato Pasquali	November 18, 2009	University Alma Mater Studiorum, Bologna, Italy	Obesity, stress, and sex differences: the role of steroids and central neuroendocrine networks
David Allison	February 3, 2010	University of Alabama at Birmingham, Birmingham, Alabama	Conjectures on some curious connections among social status, hunger, fatness, and longevity
Paolo Sassone-Corsi	January 6, 2011	University of California, Irvine, California	Mammalian circadian clock and metabolism the epigenetic link
Weihong Pan	May 19, 2011	Pennington Biomedical Research Center, Baton Rouge, Louisiana	Leptin action on non-neuronal cells in the central nervous system; potential clinical implications.
Hans Rudi Berthoud	November 10, 2011	Pennington Biomedical Research Center, Baton Rouge, Louisiana	Metabolic and hedonic drives in the neural control of appetite: who is the boss?

## Beyond thermodynamic laws

The first lecture by David Allison provided an overview, mostly based on his group's original work, on multiple putative factors that may contribute to the obesity epidemic. He set forth the interesting idea that the perceived*,* rather than the actual, amount of calories available in the foreseeable future influences feeding behavior and the willingness to spend instead of saving energy. This novel idea is rooted in robust work conducted in animals and humans and has important clinical and epidemiological implications. Specifically, perceived energetic uncertainty, whether of mere food in animals or of economic resources in humans, is likely to set into motion behavioral responses that eventually lead to increased fat storage. This is compatible with the well-known observation in industrialized societies that obesity is more prevalent in the less-privileged strata of the society. Allison also pointed out that social status, both in animals and humans, has a role in determining the amount of adiposity and its distribution (i.e., visceral versus subcutaneous). Furthermore, he reviewed the interdependence of energy intake and energy expenditure. When monkeys were calorie restricted by approximately 30%, a reduction in physical activity was observed, a clear attempt to defend the current body weight.

If stressful uncertainty inherent to a low socioeconomic status may lead to increased adiposity, then any intervention offering healthier diets and advice on proper portion size is destined to be less effective as long as the perceived or real uncertainty remains. In summary, Allison underlines the under-appreciated importance of psychological (i.e., perceived versus real food insecurity) and sociological (developing versus industrialized and post-industrialized societies) factors in determining adiposity and its distribution within an organism, as well as among members of a given society.

## Is the blood–brain barrier truly a “barrier”?

According to the Merriam-Webster Dictionary a barrier is: “something material that blocks or is intended to block passage.” Robert (Bill) Banks makes the cogent case that this term was never scientifically correct. Using leptin as an example, Banks elucidates the role of the blood–brain barrier (BBB)—this fastidious and sophisticated structure—in regulating body weight. Leptin is a large molecule, and its transport to the brain is highly regulated and facilitated by specific carriers. This regulation is not immutable over time but rather is influenced by short-term and long-term changes in physiological states. Thus, when peripheral levels of the fat-derived hormone leptin decline, as a result of decreased fat deposition and/or increased fat catabolism, the brain becomes “alerted” to the fact that times are getting more difficult. It may not be the right time to invest in reproduction or other expensive behaviors, but rather save energy.

The orchestrated series of neurobehavioral responses to feeding are the result of millions of years of evolution that have shaped the features of many different species. However, evolution is efficient but slow. Living beings are exquisitely adapted to survive in a world of limited and precarious food resources. They have not had time, however, to adapt equally efficiently to conditions in which the brain needs to know that too much fat is being accumulated, a relatively novel condition from an evolutionary standpoint. As a consequence, the BBB is not as effective in transporting leptin inside the brain when large concentrations of plasma leptin are pushing at “the gates” of the brain. In other words, the hormone leptin evolved in conditions of paucity and non-predictability of food availability.

Further interesting experimental observations summarized by Banks are the effects of triglyceride levels on the permeability of the BBB. During periods of fasting, plasma triglycerides increase as a result of mobilization of fat reserves; this may be teleologically advantageous to alert the brain that reserves are being consumed. Triglycerides apparently impair the transport of leptin into the brain. Elucidation of the structural and molecular mechanisms that allow modifications of the BBB is yet to come, but this knowledge may promote effective drug development and novel use of known drugs (such as leptin) for the treatment of obesity. On a more general note, a better understanding of the mechanisms regulating access to the brain may result in a better understanding on how to deliver therapeutic agents to the central nervous system.

## Embracing the complexity of the stress response

Renato Pasquali provided an overview of the fascinating and rapidly evolving research field of the stress response, elegantly integrating his own work with that of well-known scholars, including Biorntorp, Dallman, McEwen, and Sapolski, in addition to Chrousos and Gold. The latter two codified the now classic functional description of the stress system in their seminal review article published in *JAMA* in 1992, defining the effector branches of the stress system as the hypothalamic–pituitary–adrenal (HPA) axis and the sympatho–adrenal–system (SAS). The complexity of these systems is, however, daunting. Where does this complexity come from? First of all, characterizing the level of activity of the effectors of the stress system, the HPA axis and SNS, is elusive, as Pasquali knows well, based on his clinical experience on patients with Cushing's syndrome, Addison's syndrome, pheochromocytoma, and similar conditions. In healthy subjects, this task is even more difficult, as abnormalities are more subtle and of less obvious clinical consequence, at least in the short-term. Borrowing from the principle of uncertainty originally formulated by Werner Heisenberg, the methods we use to measure a phenomenon (in Heisenberg's case particle momentum) perturb the system; *mutatis mutandis*, even the minor stress of venipuncture increases ACTH and catecholamine levels, for example. Thus, the stress system is rapid in its reaction, making any instantaneous picture already outdated. In addition, stress, or more properly, *stressors* are wide ranging; they may consist of physical factors, such as pain and fasting, or more complex challenges, such as sleep deprivation; they may be purely psychological, such as immobilization in rodents and acute anxiety in humans, or they can—and will—resonate differently in different individuals, a concept known as *coping*. In addition, there are sex differences, genetic differences, and species-dependent differences. *Stressology*—provided such a word exists—is an eminently interdisciplinary science that includes, and benefits from, contributions of various fields, from human genetics to biostatistics, history, sociology, and many others.

In reference to the application of these general principles to the field of obesity, Pasquali summarizes the now classic observations that body fat distribution is influenced by glucocorticoids—alas stress—and that there are differences between males and females that, albeit present in humans, seem to become less accentuated as we move down the phylogenetic scale. Obesity, similar to stress, is a phenotype difficult to characterize. This is exemplified by the measure of body mass index (BMI), which lumps together individuals with very heterogenous body compositions, all being equally labeled as obese when surpassing a certain number. In a similar fashion, the definition of metabolic syndrome (MS) encompasses many heterogeneous conditions. To have MS, one needs to have at least 3 of 5 components. We will call these components A–E. Therefore, one could have MS based on three components: A + B + C, A + B + D, A + B + C, or A + B + E; four components; or five components. Subjects may be labeled as having the same condition over time but in reality they have two different conditions (A + B + C versus, as an example, C + D + E). Furthermore, the definition is categorical and does not differentiate between a person with a marginally elevated cholesterol concentration (e.g., 201 mg/dL) versus a very high concentration, which is associated with serious medical risks. We wonder about the significance of such a vague definition. There may definitely be value *tout court* for those who have commercial interests in patentable remedies, since the vast majority of living human beings, and soon our pets, will be labeled as suffering from MS.

Returning to Pasquali's review, the effects of stress on MS are also described. Among the most interesting take-home messages are the notion that the stress response varies not only interindividually but also according to gender, and the introduction of the concept of *resiliency*, namely, the identification of the factors predicting coping at an individual level.

## Of free will

Hans Rudi Berthoud reminds us that much of our actions, at least in the field of food behavior, is influenced by strong biological components, and thus may not be a reflection of our free will. The food reward response is redundant, involves many neurocircuits, and is represented within the mammalian brain at many neuroanatomical levels, from the more rudimental hindbrain, to the more recent and sophisticated areas, including the prefrontal cortex. In addition, the food reward system seems to be characterized by a unilateral plasticity, implying that more food calls for more food. Unfortunately, the reverse is not true, as indicated by the inability to comply with dietary restrictions for prolonged periods of time.

The experimental work of Berthoud's group aims at determining the effects of adiposity in rodents on the main components of the reward function, *liking* and *wanting*. Liking appears to be potentiated in obese animals, as clearly indicated by a switch in the dose–response curve of corn oil administration, with less response to the lower dose than to the higher dose of corn oil. Not surprisingly, wanting is heavily influenced by the dopaminergic system, as indicated by classic neuroimaging studies conducted in obese human subjects. The neurobiology of food seeking, especially the activation of the dopaminergic system, has prompted some to introduce the controversial concept of *food addiction*. Before officially codifying this characterization by adding it to the list of addictions in the upcoming DSM-V manual, the pros and cons should be carefully weighed. For example, defining obesity as a food addiction syndrome may inadvertently prompt an epidemic of anorexia nervosa in predisposed subjects.

## Exercising, not reading books, will make you smarter?

Work conducted in the laboratory of Mark Mattson has depicted the fascinating biology of brain-derived neurotropic factor (BDNF), which is a member of the neurotrophin family of growth factors, a discovery that led to the Nobel Prize for Rita Levi-Montalcini and Stanley Cohen in 1986. Mattson and his team are exploring the important roles of BDNF in controlling energy metabolism, cardiovascular functions, learning, and memory. BDNF and its cognate receptor TrkB are highly represented in brain areas crucial for energy metabolism, including several hypothalamic nuclei and the brain stem. BDNF is an appetite suppressant, as indicated by targeted disruption of this peptide in rodents. Conversely, fasting, especially if intermittent, increases BDNF concentrations in various brain regions, including the hippocampus, an important area for memory and learning. BDNF not only modulates energy intake; it also affects energy expenditure, as suggested by the observation that subjects with obesity and diabetes have low circulating levels of BDNF. In addition, BDNF is involved in the stress reaction; ablation of BDNF at birth makes animals over-sensitive to stress, as shown by an exaggerated rise in plasma corticosterone levels after immobilization. Furthermore, BDNF controls the vagal component of the autonomic nervous system; exercise- or fasting-induced rises in BDNF stimulate the vagal system, leading to lower heart rate and increased heart rate variability. Conversely, BDNF ablation makes animals more prone to an exaggerated sympathetic response. True to its name, a rise in BDNF, usually stimulated by exercise, improves cognitive functions in rodents in parallel with a documented increase in dendritic spine and sprouting. Mattson *et al.* conclude that BDNF has the potential to keep us sharp, lean, and smart, as long as we continue to live in an environment that is challenging because food is scarce and its availability unpredictable. Unfortunately, our modern environment is anything but that. Until our biology “catches up” with our culture, we need to modify our environments and habits in non-obesogenic ways.

## Unraveling a novel, and unexpected, neurobiology for the fat-derived hormone leptin

Wei Pan's review is complementary to Banks’ review in that it builds upon the complex regulation of the BBB to describe a novel role for leptin. It is well known that the transport of leptin into the brain is highly regulated via the availability of five locally expressed leptin receptors ObRa–e, derived by alternative splicing and belonging to the cytokine receptor superfamily. These receptors are expressed not only in areas such as the arcuate nucleus of the hypothalamus and the median eminence, where their presence is expected because of the role of leptin in energy homeostasis, but also in the dentate gyrus and CA1 of the hippocampus, where they modulate learning and memory. Surprisingly, these receptors are also found in several cerebellar layers. Leptin effects are also observed in the nucleus of the tractus solitarius and in the dorsal vagal complex, with possible implications for autonomic nervous system (ANS) activities, including feeding and gastric motility. Some of the central functions of leptin, as they interrelate with circadian rhythms and sleep functions, are also described in Pan's article.

The distribution of the ObRs within different CNS cells is interesting; for example, they are present in astrocytes at a lower level (∼ 20%) compared with the arcuate nucleus, and the concentration seems to be inducible, as it increases in mice with adult-onset obesity. Additional central functions of leptin include modulation of the threshold for seizures.

## To fidget or not to fidget

Catherine Kotz and her colleagues provide an interesting summary of their preclinical work on *spontaneous physical activity* (SPA)—a term that refers to undirected movements in rodents and fidgeting in humans. It is known from work of Ravussin and other groups that SPA may account for a substantial portion of total energy expenditure, ranging in humans from 100 to up to 700 calories a day. SPA has a clear genetic component and is modulated at a central level by several neuropeptides, including orexin, cholecystokinin, CRH, neuromedin, and other peptides. Specifically, the neurons that produce the two closely related peptides, orexin A and orexin B, are mostly located in a small brain area within the lateral hypothalamus. These neurons, however, have widespread projections to the rest of the brain and thus modulate fundamental functions, such as energy homeostasis, reward reactions, stress responses, and, last but not least, sleep and arousal. Overall, the main role of orexin is to expend energy and to modulate food intake and macronutrient preferences. Consistent with the above, obesity-resistant rats have increased levels of SPA in the setting of increased activity of orexin A.

Another fundamental function of orexin is the modulation of sleep, a function underlined by the neuroanatomical connections between the endogenous clock, the suprachiasmatic nucleus, and orexin cell bodies. This function is intertwined with the role in energy homeostasis and supported by the observation that subjects with narcolepsy tend to be overweight and by an animal model of narcolepsy develops early-onset obesity. The neural networks underlying SPA represent promising targets for neuropharmacological agents of potential therapeutic value in the treatment of obesity.

## The white fat–mineralocorticoid axis: a new endocrine axis?

Marco Boscaro presents preclinical and clinical evidence supporting the recent hypothesis that fat tissue and the mineralocorticoid axis cross talk. Until the discovery of leptin in 1994, fat tissue was merely regarded as a depot organ, but has now been reclassified as an endocrine organ, with “full dignity.” In addition to leptin, this organ secretes a series of cytokines and other substances that function as hormones. Boscaro highlights different mechanisms and anatomical levels by which the stress reaction of the HPA axis may differ from one individual to another. Some of these mechanisms include the role of glucocorticoid receptor polymorphisms in the individual variability to glucorticorticoid action, as well as the differential regulation of the two isoenzymes (11-β-hydroxysteroid dehydrogenase type 1 and 2) that convert cortisol into cortisone, and vice versa. Of interest, 11-β-HSD1 is present in adipocytes, where its activity is influenced by energy metabolism.

Subjects with obesity, especially the visceral type, have an increased activity of the renin–angiotensin–aldosterone system (RAAS). In turn, increased RAAS prevents further differentiation of pre-adypocytes into more mature cells; thus, these cells grow in size, becoming larger than usual and secrete a greater amount of inflammatory cytokines. The pharmacological blockade of RAAS activity, induced by angiotensin converting enzyme (ACE) inhibitors and angiotensin receptor blockers (ARB), has been associated with an improvement in insulin resistance, possibly mediated by a facilitation of adipocyte differentiation. The idea that RAAS is involved in adipocyte differentiation paves the way for pharmacological manipulation of this system with the aim of exercising anti-adipogenetic activity. In addition, Boscaro hypothesizes the presence of a factor of adipocyte origin that would, in turn, stimulate the production of aldosterone, as indicated by *in vitro* studies of adrenal cells in culture exposed to fat cell–conditioned medium.

The clinical correlate to these preclinical observations consists in subjects with primary hyperaldosteronism who have decreased insulin sensitivity compared to matched controls, and increased expression of interleukin 6 (IL-6; a diabetogenic factor) in their white adipocytes. In summary, Boscaro eloquently describes the existence of a novel, bidirectional white fat–mineralocorticoid axis.

## From complexity back to simplicity: links between chronobiology and molecular biology

It is well known that the internal pacemaker in mammals is represented by a small number of neurons, approximately 15,000, localized in the anterior hypothalamus, an area known as the suprachiasmatic nucleus. The work of Paolo Sassone-Corsi embraces the complexity of the regulation of the metabolic process at the level of peripheral tissue, and how the central clock, entrained by environmental signals including light and temperature, coordinates these processes.

The surprising observation that one out of 10 genes displays a circadian oscillation underlines the importance of chronobiology. The CLOCK (circadian locomotor output cycles kaput) gene (*Clock*) was discovered by Turek's group in 1994, the same year in which leptin was identified. *Clock* encodes for a basic helix-loop-helix-PAS transcription factor that affects both the persistence and period of circadian rhythms. CLOCK functions as an essential activator of downstream elements in the pathway critical to the generation of circadian rhythms. In their review, Sassone-Corsi and colleagues focus on how CLOCK and another core transcription factor BMAL1 (brain and muscle aryl hydrocarbon receptor nuclear translocator-like) regulate other genes, and simultaneously induce the synthesis of their own repressors, period (PER) and cryptochrome (CRY). Furthermore, they describe how CLOCK is able to induce chromatin remodeling, acting as an enzyme that opens the structure of chromatin and exposes it to transcriptional regulation. These activities require energy; and thus, the levels of nicotinamide adenine dinucleotide, (NAD^+^), a coenzyme found in all living cells, exhibit circadian oscillations. In addition, sirtuin 1 (silent mating type information regulation 2 homolog (SIRT1 in human)), is an NAD-dependent deacetylase that targets proteins that contribute to important cell processes such as reaction to stress and cell survival (e.g., reaction to stressors, longevity). The expression of nicotinamide phosphoribosyltransferase (NAMPT), the rate-limiting step for the biosynthesis of NAD^+^, is indirectly controlled by SIRT1. In summary, circadian clocks, energy, metabolism, and cell survival are intimately connected via direct molecular coupling.

## Sleep deprivation, obesity, and insulin resistance – it all adds up

Eliane Lucassen and colleagues report on the mechanistic and epidemiological work conducted by many investigators in the last two decades, including the group of Eve Van Cauter. Epidemiological evidence indicates an association between short sleep/poor sleep quality and increased weight. Lucassen also reviews in detail the neuroendocrinology of sleep, that is, the circadian changes displayed by several hormones involved in metabolism, appetite, and energy expenditure. Increased levels of proinflammatory cytokines, as indicated by the work of Alexandros N. Vgontas and colleagues, are associated with sleep deprivation; in turn, elevated levels of IL-6 and other cytokines induce sleepiness and the “sick behavior” syndrome. Finally, the article highlights the need for future studies, prospective and interventional in nature, while underlying challenges of the classic approach implementing randomized, controlled trials that may prove inept in this field.

## Sleep deprivation and obesity in children – our grandmother was right

David Gozal and colleagues describe the self-reverberating mechanisms that potentiate obesity and sleep apnea in children. The issues of obesity and sleep deprivation are qualitatively similar but quantitatively different in children and adults. Similar to adults, childhood obesity and sleep deprivation have reached epidemic proportions, but the clinical consequences and social costs are more serious. Children are more susceptible than adults (impact on growth and intellectual development), and due to their longer life expectancy, there is a longer duration of the negative consequences. Societal changes—a forced modernization—imposed on us by the mass media, including greater use of TV, especially in the bedroom, use of mobile phones, and other electronic media play an important role. Though children have a greater need for more sleep than adults, the exact amount is variable and cannot be directly determined. Lack of proper family routines regarding meals, bedtime, and regular exercise make things even worse, especially because they often result in a lifetime lack of healthy habits, which may then affect the next generation. In conclusion, Gozal argues, and we strongly agree, that sufficient knowledge has accumulated, both from the point of view of biological plausibility and epidemiological evidence, to inform health policies.

The Brain and Obesity Lecture Series was fully supported by the National Institutes of Health (NIH), Intramural Research Program: National Institute of Diabetes and Digestive and Kidney Diseases (NIDDK). We would like to thank the speakers for their contributions. We would also like to thank the staff of *Annals of the New York Academy of Sciences* and the New York Academy of Sciences for their support in preparing this publication.

